# Asia: Changing Times and Changing Problems

**DOI:** 10.1289/ehp.11856

**Published:** 2008-09

**Authors:** Frank E. Speizer, Aaron Cohen, Sumi Mehta

**Affiliations:** Channing Laboratory, Harvard Medical School, Boston, Massachusetts, E-mail: frank.speizer@channing.harvard.edu; Health Effects Institute, Boston, Massachusetts

Asia is currently experiencing rapid increases in industrialization, urbanization, and vehicularization. As a result, emission trends (e.g., energy, fuel, vehicle use), population trends (e.g., degree of urbanization, urban population growth, city size), health trends (e.g., age structure, background disease rates), and other important factors (e.g., broad changes in regulatory approaches, improvements in control technology) will influence the extent to which exposure to air pollution affects the health of the Asian population over the next several decades. Because the effects on air quality of recent, rapid development are clearly apparent in many of Asia’s cities and industrial areas, government decision makers, the private sector, and other local stakeholders are increasingly raising concerns about the health impacts of urban air pollution. Major Asian cities, such as Shanghai (China), Delhi (India), Ho Chi Minh City (Vietnam), and Manila (Philippines), now experience annual average levels of respirable particles [particulate matter ≤ 10 μm in aerodynamic diameter (PM_10_)] in excess of the World Health Organization’s (WHO) newly revised world air quality guideline of 50 μg/m^3^ (WHO 2006).

The health impacts in the region are already estimated to be substantial. The WHO (2002) estimated that urban air pollution contributed to approximately 800,000 deaths and 6.4 million lost life-years worldwide in 2000, with two-thirds of these losses occurring in rapidly urbanizing countries of Asia. These estimates were made using the results of U.S. studies of long-term exposure to air pollution because such studies have not yet been conducted in the developing countries of Asia, where health, health care, exposure to pollution, and socioeconomic circumstances still differ markedly from the United States. This contributes considerable uncertainty to these and other recent estimates of health impacts of air pollution (Cohen et al. 2004).

High-quality, credible science from locally relevant studies is essential to address the substantial air pollution challenges in Asia. Such studies will be critical in helping decision makers decide which policies are most likely to result in public health benefits. Although the number of published studies on the health effects of air pollution in Asia has grown nearly exponentially over the past quarter century, with > 400 reports in the peer-reviewed literature [Health Effects Institute (HEI) 2008], few coordinated, multicity time-series studies have been conducted comparable to the robust and consistent results in the United States and Europe (Katsouyanni et al. 2001; Samet et al. 2000). The Public Health and Air Pollution in Asia (PAPA) studies in Hong Kong, Shanghai, and Wuhan, China, and Bangkok, Thailand, published in this issue of *Environmental Health Perspectives* (Kan et al. 2008; Qian et al. 2008; Vichit-Vadakan et al. 2008; Wong et al. 2008a, 2008b), comprise the first coordinated multicity analyses of air pollution and daily mortality in Asia. These studies, designed and conducted by local investigators in concert with local air pollution and public health officials and international experts, explored key aspects of the epidemiology of exposure to air pollution in each location, providing additional insight about how factors such as weather (particularly high temperatures) and social class might modify the air pollution relative risk. Although clearly relevant to contemporary Asian conditions, these results also have global relevance.

The studies were conducted using the same types of mortality and air pollution data used in time-series studies throughout the world, and with methodologic rigor that matches or exceeds that of most published studies, including formal quality control in the form of detailed standard operating procedures for data collection and analysis, and external quality assurance audits of the data overseen by the funding organization. These studies also benefited from recent efforts to strengthen and refine methods for the analysis of time-series data; as a result they are on a par methodologically with the most recent U.S. and European analyses (HEI 2003).

These five studies provide a relatively consistent, if limited, picture of the acute mortality impact of current ambient particulate air pollution in several large metropolitan areas in East and Southeast Asia. Wong et al. (2008b) report that a 10-μg/m^3^ increase in PM_10_ level was associated with a 0.6% (95% confidence interval, 0.3–0.9) increase in daily rates of all natural-cause mortality, estimates comparable to or greater than those reported in U.S. and European multi-city studies. Interestingly, these proportional increases in mortality are seen at levels of exposure several times higher than those in most large Western cities (mean levels, 51.6–141.8 μg/m^3^), and in each city except Shanghai, the pattern of the exposure–response functions appear linear over a fairly large range of ambient concentrations up to and sometimes > 100 μg/m^3^.

Although only four cities were studied, these results may begin to allay concerns regarding the generalizability of the results of the substantial, but largely Western, literature on the effects of short-term exposure to air pollution. The results, which are broadly consistent with previous research (HEI 2004), suggest that neither genetic factors nor longer-term exposure to highly polluted air substantially modify the effect of short-term exposure on daily mortality rates in major cities in developing Asia. This provides support for the notion, implicit in the approach taken in the WHO’s world air quality guidelines (Krzyzanowski and Cohen 2008), that incremental improvements in air quality would be expected to improve health, even in areas with relatively high ambient concentrations.

Health impacts in cities in developing countries of Asia result from exposures to a mixture of pollutants, particles, and gases, which are derived in large measure from combustion sources (Harrison 2006; Wong et al. 2008b). This is, of course, no different from in Europe and North America, but the specific sources and their proportional contributions are different, with open burning of biomass and solid waste materials, combustion of lower-quality fuels including coal, and two- and three-wheeled vehicles contributing a larger share in Asia. Time–activity patterns, building characteristics, and proximity of susceptible populations to pollution sources also differ in ways that may affect human exposure and health effects (Janssen and Mehta 2006). Our current knowledge of these issues is rudimentary, and additional research is clearly needed to inform effective and sustainable control strategies. From past experience in the West and current evidence in Asia, substantial increases in the combustion of fossil fuels for power generation and transportation in developing Asia will have important consequences for human health and environmental quality in Asia and beyond. Effective approaches to pollution control and reduction do exist, and investment in these approaches need not necessarily impede economic growth. Therefore, developing countries of Asia may be able to avoid increased environmental degradation and associated health impacts while reducing poverty and providing economic security for their populations (Center for Science and the Environment 2006).

Thirty million people currently live in the four cities studied, so even the small proportional increases in daily mortality rates imply large numbers of excess deaths. That said, air pollution is but one of many factors that affect the health of people in developing Asia, and, unfortunately, not even the most important one (Ezzati et al. 2002). Nonetheless, the substantial health impacts of exposure to air pollution should be of concern to public health policy makers faced with difficult decisions in transportation and energy policy. Given current predictions of even more accelerated urbanization in the regions, there will be an increasing need for more extensive monitoring of urban air quality designed to support health effects studies and impact assessments, and a corresponding need for more highly trained professionals in air quality monitoring, exposure assessment, and environmental epidemiology.

Strategic planning for future research is also needed. Although our ability to draw firm conclusions from results in four cities is limited, the methods of Wong et al. (2008b) can be replicated in additional cities across the regions. In some cases, nonmortality outcomes, such as hospital admissions, may also be addressed, enabling policy makers to better quantify the health impacts of air pollution. However, while time-series studies such as the PAPA studies will continue to be important potential drivers of environmental and public policy, additional study designs, such as cohort studies—similar to the U.S. American Cancer Society (Pope et al. 2002) and Six Cities (Laden et al. 2006) studies—are needed in Asian populations to estimate effects of long-term exposure on annual average mortality and life expectancy, the metrics that may be the most meaningful and policy relevant to decision makers. These kinds of studies will require the building of multidisciplinary teams of investigators, with adequate long-term commitment of resources to work in collaboration with governmental officials, their industrial counterparts, and local stakeholders. The PAPA program is one model of how such resources can be brought together to support such an effort.

## Figures and Tables

**Figure:**
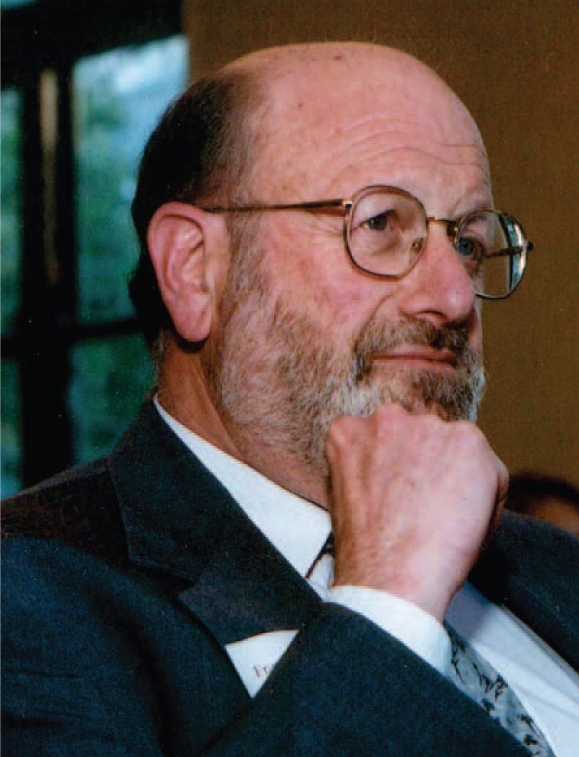
Frank E. Speizer

**Figure:**
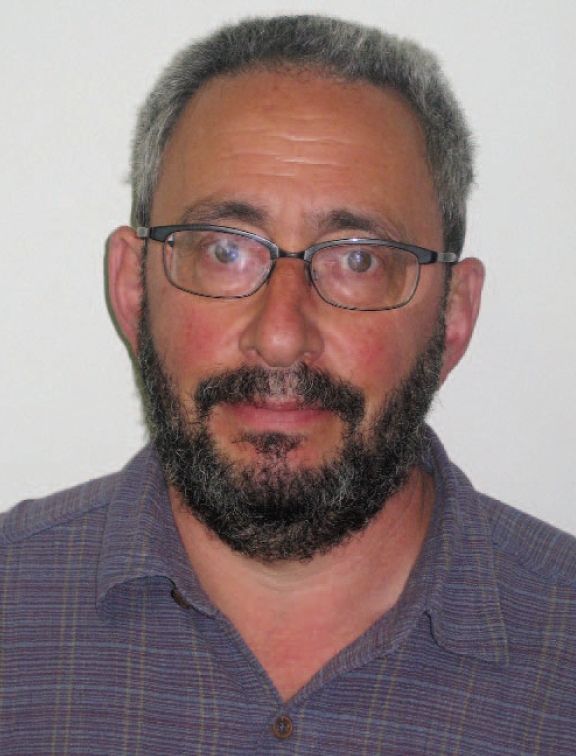
Aaron Cohen

**Figure:**
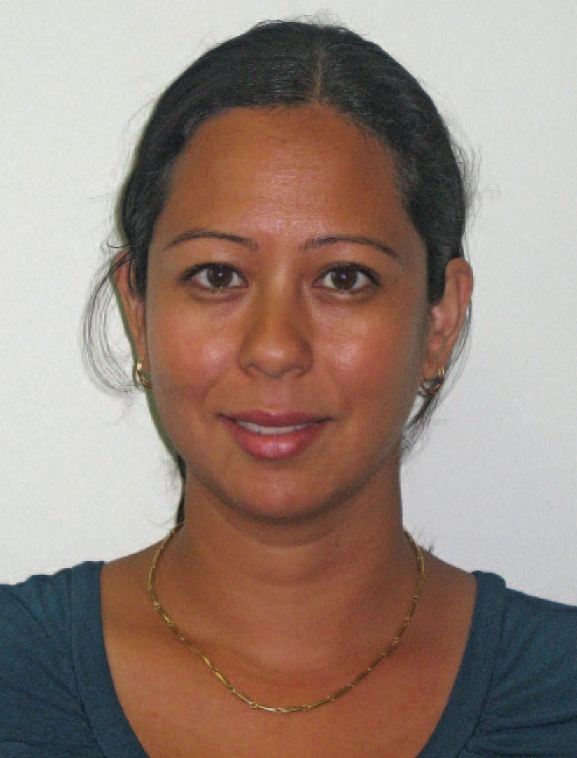
Sumi Mehta
